# Serum Interleukins 8, 17, and 33 as Potential Biomarkers of Colon Cancer

**DOI:** 10.3390/cancers16040745

**Published:** 2024-02-10

**Authors:** Constantin-Dan Tâlvan, Liviuța Budișan, Elena-Teodora Tâlvan, Valentin Grecu, Oana Zănoagă, Cosmin Mihalache, Victor Cristea, Ioana Berindan-Neagoe, Călin Ilie Mohor

**Affiliations:** 1 Faculty of Medicine, “Lucian Blaga” University of Sibiu, 550169 Sibiu, Romania; talvan_dan@yahoo.com (C.-D.T.); cosmin.mihalache@ulbsibiu.ro (C.M.); calin.mohor@ulbsibiu.ro (C.I.M.); 2 Research Center for Functional Genomic, Biomedicine and Translational Medicine, “Iuliu Hațieganu” University of Medicine and Pharmacy Cluj-Napoca, 400012 Cluj-Napoca, Romania; liviuta.petrisor@umfcluj.ro (L.B.); oana.zanoaga@umfcluj.ro (O.Z.); victor_cristea@yahoo.com (V.C.); ioananeagoe29@gmail.com (I.B.-N.); 3 Faculty of Engineering, “Lucian Blaga” University of Sibiu, 550025 Sibiu, Romania; valentin.grecu@ulbsibiu.ro

**Keywords:** colon cancer, interleukins, serum, biomarkers, cancer staging, age, gender, correlations

## Abstract

**Simple Summary:**

This research studied how three types of proteins, called interleukins (namely 8, 17A and 33), are present in the blood of healthy people and people with colon cancer. Colon cancer is a disease that affects the large intestine. The paper looked at 82 people, 42 of whom had colon cancer and 40 of whom did not. The researchers divided the cancer patients into four groups based on how severe their cancer was. The study measured the amount of interleukins in the blood of each person using a special test. The paper analyzed the results of the test, considering the age, gender, and cancer stage of each person. The results showed that younger people, and those with less severe cancer had more interleukins in their blood. The paper also found that two of the interleukins (8 and 17A) were higher in the cancer group, while one of them (33) was higher in the healthy group. The study also found that the interleukins were related to each other in both groups. This research concluded that the interleukins might help detect colon cancer and predict how it will progress. This paper could help improve the diagnosis and treatment of colon cancer.

**Abstract:**

This research investigated the serum levels of three interleukins (IL8, IL17A, and IL33) and the possible relationships between them in healthy people and colon cancer patients at different stages. This study involved 82 participants, 42 of whom had colon cancer and 40 were healthy individuals. The cancer patients were classified into four groups according to the TNM staging classification of colon and rectal cancer. Serum levels of the interleukins were measured by the ELISA test. The data were analyzed statistically to compare the demographic characteristics, the interleukin levels across cancer stages, and the correlation between interleukins in both groups. The results showed that women had more early-stage colon cancer diagnoses, while men had more advanced-stage cancer diagnoses. Stage two colon cancer was more common in older people. Younger people, men, and those with early-stage colon cancer had higher levels of interleukins. The levels of IL8 and IL17A were higher in the cancer group, while the level of IL33 was higher in the healthy group. There was a strong correlation between IL8 and IL17A levels in both groups (*p* = 0.001). IL17A influenced the level of IL33 in the cancer group (*p* = 0.007). This study suggested that cytokine variation profiles could be useful for detecting colon cancer and predicting its outcome.

## 1. Introduction

Colon cancer is considered to be the main cause of death from gastrointestinal malignancies [1] and the second most deadly among cancers. Although its incidence has generally decreased over the last 20 years, it is increasingly present among patients under 50 years of age [2]. Various studies have shown that countries with the highest HDI score have a decrease in colon cancer incidence [3]. Diagnosis in the early stages of colon cancer through biomarker detection is important, as advanced metastasis complicates treatment and lowers survival rates [4]. Studies of age related to colon cancer progression have shown low survival rates with increasing age [5]. Although colon cancer is proven to be genetically determined and influenced by epigenetic factors involving lifestyle such as smoking, alcohol, lack of activity, presence of polyps, radiation, or advanced age, studies have shown a higher incidence of colon cancer in urban areas than in rural ones [6]. Overall, possibilities of screening and high quality treatment are highly associated with higher income countries [7].

Inflammation is an important risk factor for colon cancer development and proinflammatory cytokines can influence colorectal cancer development [8,9]. The NLRP3 inflammasome is a complex of proteins that responds to various signals and triggers the release of inflammatory cytokines, such as IL-1β and IL-18, and the death of cells by pyroptosis, which causes more inflammation [10,11]. The NLRP3 inflammasome is controlled by different pathways, one of which is the MyD88-dependent pathway, which is activated by TLRs when they detect PAMPs. This pathway stimulates NF-κB and MAPKs, which increase the expression and activity of NLRP3 and pro-IL-1β by modifying them [12]. The NLRP3 inflammasome is involved in many diseases, such as cancer and cardiovascular diseases. It can have opposite effects on cancer, depending on the type, stage, environment, and immunity of the tumour. It can either help or hinder colon cancer by affecting inflammation, blood vessels, invasion, spread, immunity, and cell death [10,11]. It can also influence the outcome of cancer therapy, such as immune checkpoint inhibitors, by changing the levels of inflammatory and anti-inflammatory cytokines [12]. A dysfunctional immune system and aberrant expression of some cytokines can determine colon cancer onset and progression [13]. Interleukin 8 is an important proinflammatory cytokine upregulated in different malignancies due to the fact that it is controlled by a nuclear factor (NFkB), which is activated in cancer and inflammation [14]. Circulating interleukin 8 has been shown to be a strong prognostic factor for colorectal cancer [15]. A possible tumour-promoting role of interleukin 8 has been suggested in colon cancer [16], as several studies have shown an upregulation of IL8 in tumour progression and development of colon cancer [1]. Interleukin 17 a is a member of the Th17 cell subset and has an important role in autoimmunity, cancer, and inflammation [17]. Interleukin 17A is widely known to be a promoter of colon cancer initiation and progression [18]. Several studies have shown the involvement of IL 17 in colorectal cancer metastasis and prognosis and it is strongly associated with poor outcomes of this malignancy [19]. Studies have also shown high levels of IL17A-producing cells in patients with poor colorectal carcinoma prognosis [20]. Interleukin 33 is a cytokine of the IL1 family that is expressed in inflammation and trauma by being released upon tissue injury as an alarm cell in the immune system [21,22]. IL-1 is an inflammatory cytokine that affects both colorectal cancer and cardiovascular diseases. It can stimulate tumour progression and cardiac damage, as well as interfere with cancer therapy. IL-1 inhibitors, such as canakinumab, are antibodies that prevent IL-1 from binding to its receptor and lowering inflammation. Canakinumab could benefit colorectal cancer patients, especially those with cardiovascular problems or who are receiving anticancer drugs. Canakinumab could protect the heart from toxicity and improve the response to therapy by altering the tumour and immune environment [23]. It has been shown that IL33 can promote metastasis of colorectal cancer in mice by being activated by proinflammatory cytokines released in tumour microenvironments [24]. However, the exact role of IL33 is yet to be determined as the literature also provides studies that show an antiproliferative effect of this interleukin and an inhibition of colon cancer growth [25]. Furthermore, IL33 may act as a promoter or inhibitor of colorectal cancer tumourigenesis depending on the specific cancer subtype [26].

The study of colon cancer has been significantly enriched by the understanding of the roles of specific interleukins, particularly IL8, IL17A, and IL33. These interleukins are known to play crucial roles in inflammation and tumour progression [27]. IL17A, for instance, is a potent proinflammatory cytokine that contributes significantly to the formation, growth, and metastasis of a wide range of malignancies, including colon cancer [27]. Similarly, IL33 plays a role in tumour immune escape in cancers via Th2 cells and regulatory T cells. Given their importance, this paper aims to evaluate possible correlations between these three interleukins and assess whether these correlations could regulate colon cancer progression. By doing so, we hope to contribute to the growing body of knowledge on the pathogenesis of colon cancer and potentially inform future therapeutic strategies.

The purpose of this study was to evaluate IL8, IL17, and IL33 concentrations in healthy and colon cancer patients and their variation in different stages of this malignancy and to establish the existence of any correlations between these three interleukins in colon cancer progression.

## 2. Materials and Methods

### 2.1. Study Population

This study was carried out on 42 colon cancer patients who underwent surgery and 40 control subjects scheduled for routine colonoscopy. Cancer patients were divided into 4 groups after being selected from the department of General Surgery of Sibiu University Hospital over a period of 6 months. Cancer subjects (n = 42, 19 males and 23 females with ages ranging from 45 to 86 years, with a mean age of 68 years) were divided into 4 groups according to the TNM staging classification of colon and rectal cancer. The TNM staging classification of colon and rectal cancer is a system that describes the extent and spread of the disease based on three factors: T (tumour), N (node), and M (metastasis) [28]. The tumour (T) factor indicates how far the cancer has grown into the wall of the colon or rectum, which consists of several layers, from the inner mucosa to the outer serosa. The node (N) factor reflects whether the cancer has reached nearby lymph nodes, which are small organs that filter lymph fluid and help fight infections. The metastasis (M) factor shows whether the cancer has spread to distant organs, such as the liver or lungs, or to the peritoneum, which is the tissue lining the abdomen. Each factor is assigned a number or letter to indicate the severity of the cancer, with higher numbers or letters meaning more advanced disease. For example, T1 means the tumour is confined to the inner layer of the colon or rectum, while T4b means the tumour has invaded adjacent organs. The TNM factors are then combined to assign an overall stage, from stage 0 (the earliest stage) to stage IV (the most advanced stage). The TNM staging system helps doctors determine the prognosis and treatment options for colorectal cancer patients [29].

The purpose of this study was fully explained to each subject before entering the study and informed consent was obtained after a complete medical examination. Inclusion criteria for cancer subjects were colorectal adenocarcinoma with histopathological confirmation of disease stage, no prior chemo- or radiation therapy, and adequate performance status of the patient. Exclusion criteria were inflammatory diseases that could have influenced cytokine serum levels, treatments with effects of immune response (such as chemotherapy, cortisol, and others), other active infections or malignancies, cardiovascular events over the last 6 months, and severe organ deficiencies or injuries.

### 2.2. Collection of Blood Samples

Venous blood samples, from the median cubital vein, were collected from each subject in 2 mL vacutainer glass tubes for coagulation. Two tubes were collected from each patient. The samples were centrifuged after clotting for 10 min at 4200 rpm. The obtained blood serum was place into sterile Eppendorf vials and refrigerated at −80 °C until being assayed.

### 2.3. Biochemical Analysis

The ELISA kits were purchased from BioLegend (San Diego, CA, USA) and were used for quantification of the proteins in the blood samples. In the present study, the ELISA kits used were as follows: LEGEND MAX™ Human IL-33 ELISA Kit (cat.no. 435907), LEGEND MAX™ Human IL-8 ELISA Kit (cat. no. 431507), and LEGEND MAX™ Human IL-17A ELISA Kit (cat.no. 433917). BioLegend’s LEGEND MAX™ ELISA Kits are a sandwich enzyme-linked immunosorbent assay (ELISA) with a 96-well strip plate that is precoated with a capture antibody. The kits are specifically designed for the accurate protein quantitation of human IL-33, human IL-17A, and human IL-8 from serum samples used in our study. Serum levels of IL-33, IL-17A, and IL-8 were determined by ELISA according to the manufacturer’s instructions (BioLegend, San Diego, CA, USA). Concentrations were calculated by comparison with standard curves. For more accurate results, by avoiding interaction between analytes during the testing process, molecules were tested individually and not by multiplexed analysis. A Biotek Synergy H1 hybrid absorbance microplate reader was used to perform ELISA testing on interleukins 8, 17A, and 33.

### 2.4. Statistical Analysis

The statistical analysis of the data collected for the research on serum interleukin 8, 17, and 33 as potential biomarkers of colon cancer was performed as follows. First, the data were organised and cleaned using Microsoft Excel, and then imported to Minitab 20 software for further analysis. Second, descriptive statistics such as mean, standard deviation, minimum, maximum, and median were calculated for each variable and presented in a table. Third, graphical analysis such as boxplots and histograms were used to visualise the distribution and variation of the data and to identify any outliers or skewness. Fourth, multiple regression analysis was used to examine the relationship between the serum levels of interleukin 8, 17, and 33 and the colon cancer status, controlling for other factors such as age, gender, and stage of the disease. The regression coefficients, standard errors, *p*-values, and R-squared were reported and interpreted. Fifth, 2-sample *t* tests were conducted to compare the mean values of interleukin 8, 17, and 33 between healthy and cancer patients, assuming equal variances. The t-statistics, degrees of freedom, *p*-values, and confidence intervals were reported and interpreted. The level of significance was set at 0.05 for all tests, which means that we would reject the null hypothesis if the *p*-value was less than or equal to 0.05.

## 3. Results

### 3.1. Descriptive Analysis

**Figure 1:**
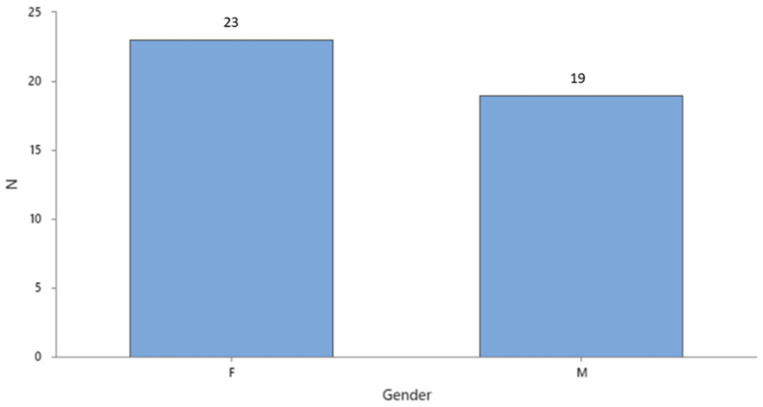
Gender distribution of colon cancer subjects.

Gender distribution of colon cancer patients involved in this study shows a slightly higher incidence of this malignancy in women (see Figure 1).

**Figure 2:**
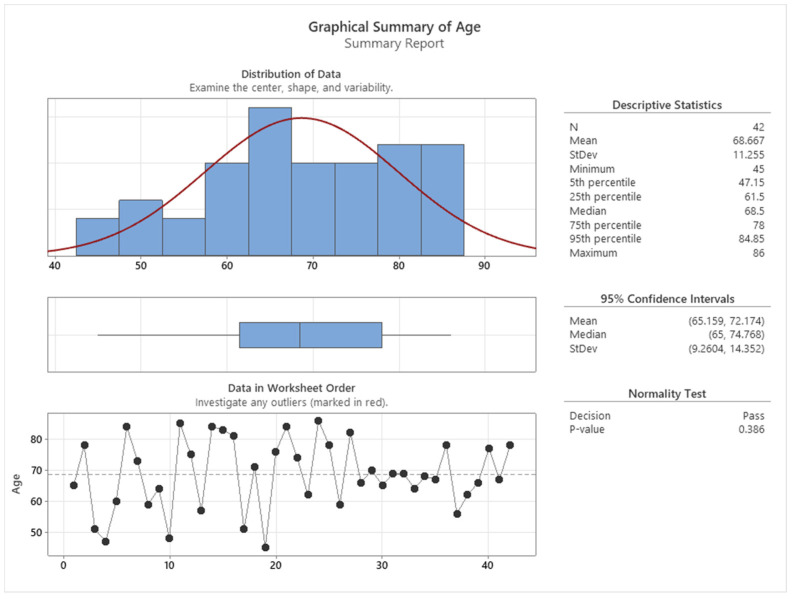
Age distribution of colon cancer subjects.

Figure 2 shows that the cancer patients who participated in this study were aged between 45 and 86 years old and their age distribution passes the normality test, with a mean of 68.6, indicating that the highest incidence of colon cancer is observed at the age of 68 years.

**Figure 3:**
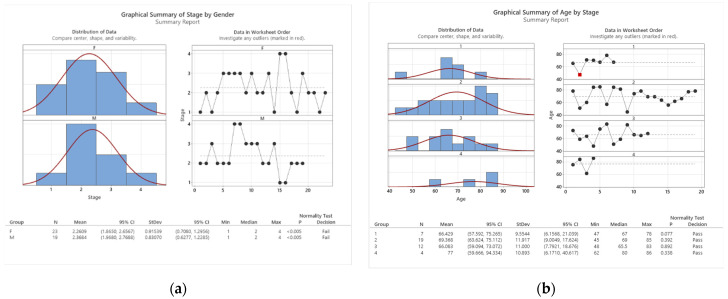
Colon cancer staging by gender (**a**) and age (**b**).

We analyzed the distribution of the four cancer stages among the patients who participated in this study. We found that the distribution followed a normal curve for both gender (Figure 3a) and age (Figure 3b) variables. This means that the majority of the patients were in stage 2 or 3, while the minority were in stage 1 or 4. We discuss the implications of this finding in the next section.

**Figure 4:**
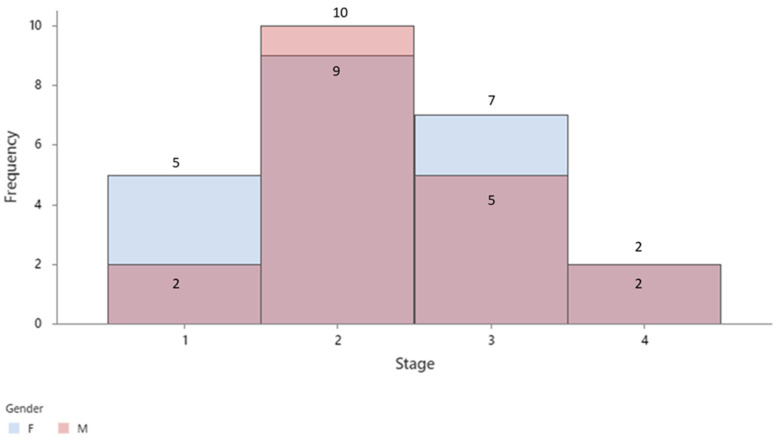
Colon cancer staging by stage and gender.

As shown in Figure 4, the stage of colon cancer was associated with both gender and age of the patients. Specifically, males had a higher prevalence of stage 2 colon cancer than females, and older patients were more likely to have stage 2 colon cancer than younger patients.

**Figure 5:**
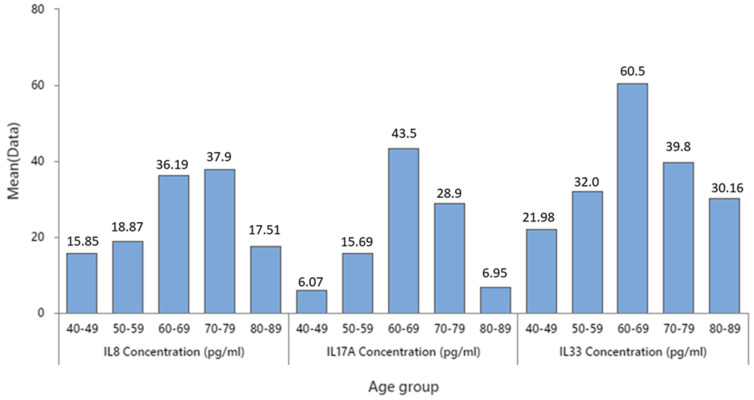
IL 8, 17, and 33 mean concentration by age groups.

Interleukin mean concentrations in different age groups show the highest concentration of IL8 in the 70–79 age group, followed by the 60–69 age group with a slightly lower concentration. The highest concentration of IL17A and IL33 was in the 60–69 age group of colon cancer subjects, with significant difference in these IL concentrations between the 60–69 age group and the other age groups (see Figure 5 and Table 1).

**Figure 6:**
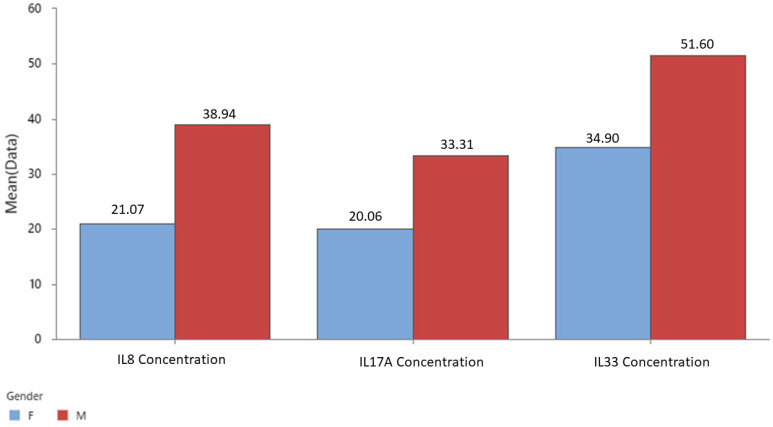
Mean concentration of IL 8, 17, and 33 by gender.

Figure 6 and Table 2 illustrate the average levels of the three inflammatory cytokines, namely IL8, IL17, and IL33, in male and female subjects. The results indicate that males have significantly higher concentrations of these cytokines than females, suggesting a gender difference in the immune response to the malignancy.

**Figure 7:**
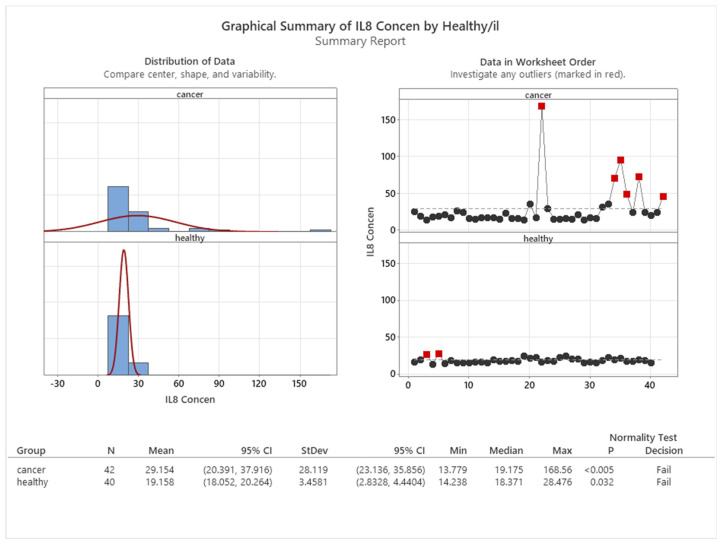
Mean concentration of IL8 in healthy and cancer patients.

As shown in Figure 7, the control group of healthy subjects had significantly lower interleukin 8 expression, with a mean value of 19.158, compared with the group of cancer patients, whose mean value was 29.154.

**Figure 8:**
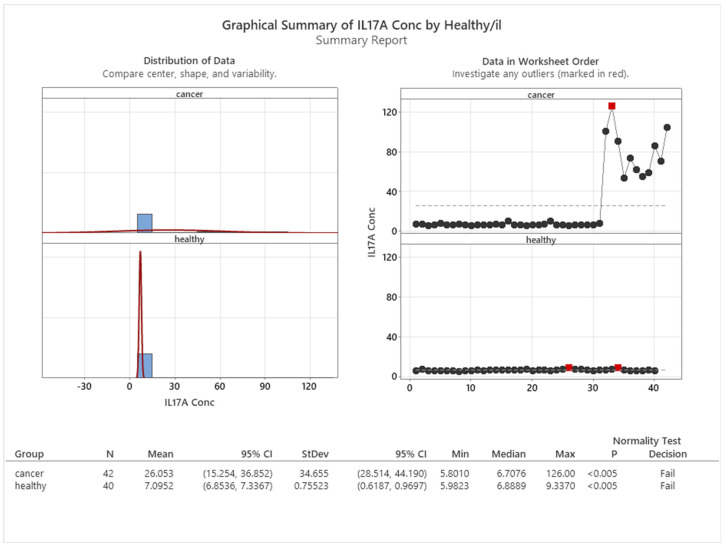
Mean concentration of IL17A in healthy and cancer patients.

One of the factors that distinguishes cancer patients from healthy individuals is the level of IL17A in their blood. Figure 8 shows the mean IL17A concentration for both groups, measured in pg/mL. The cancer group had a significantly higher mean value of 26.053 pg/mL, indicating an elevated inflammatory state. The control group, composed of healthy participants, had a much lower mean value of 7.095 pg/mL, suggesting a normal immune function.

**Figure 9:**
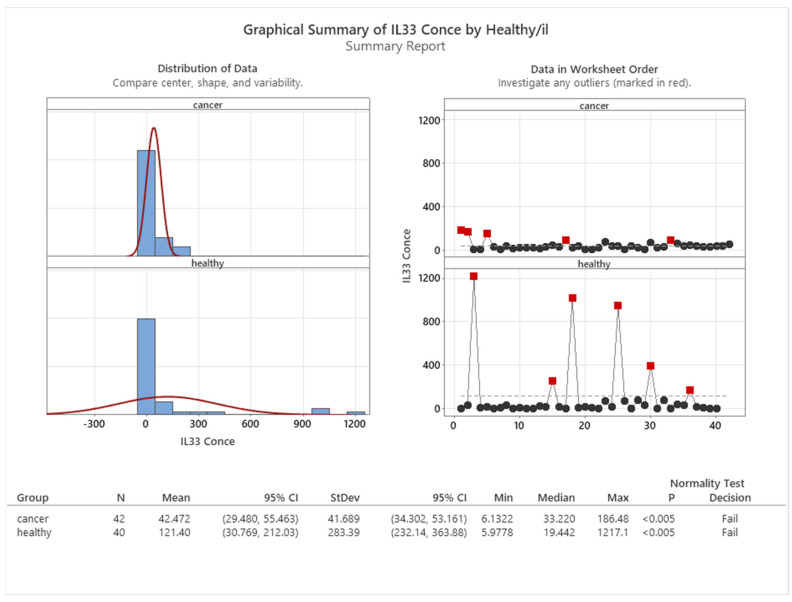
Mean concentration of IL33 in healthy and cancer patients.

In this study, we measured the concentration of IL33 in the serum of cancer patients and healthy controls. We found a significant difference between the two groups, with healthy controls having a higher mean concentration of IL33 (121.40 pg/mL) than cancer patients (42.47 pg/mL). This difference is shown in Figure 9, which displays the distribution of IL33 levels in both groups.

**Figure 10:**
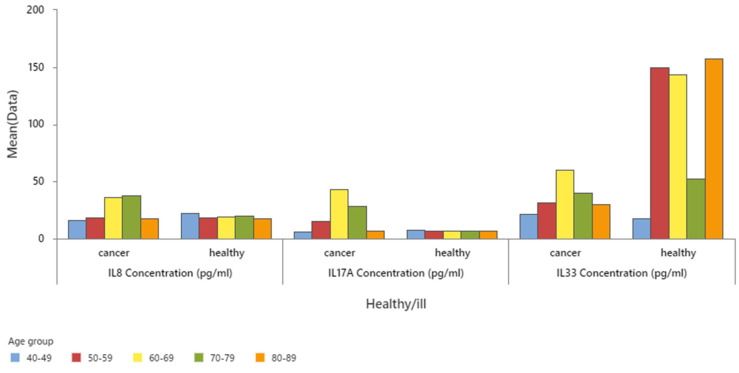
Mean concentrations of IL8, 17A, and 33 in healthy and colon cancer staging.

The levels of interleukin 8 (IL8) and interleukin 17A (IL17A) were measured in the serum samples of cancer patients and healthy individuals. The results showed that cancer patients had significantly higher concentrations of IL8 and IL17A than the control group, indicating a proinflammatory state associated with the tumour stage. On the other hand, the concentration of interleukin 33 (IL33) was higher in the healthy group than in the cancer group, suggesting a protective role of this cytokine against cancer development. These findings are illustrated in Figure 10 and presented in Table 3 and Table 4.

**Figure 11:**
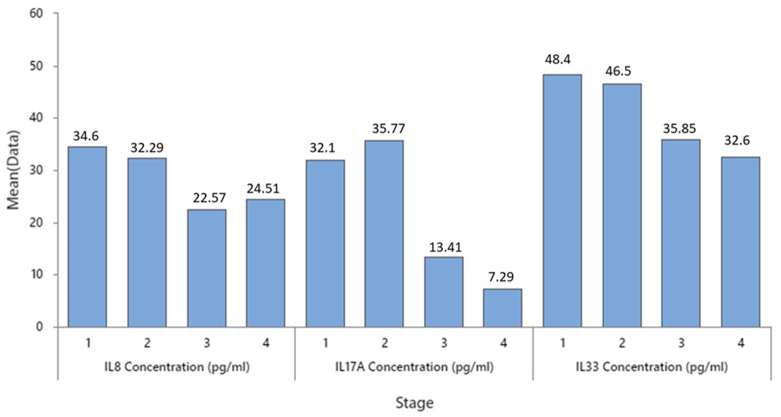
Mean concentration of IL8, 17, and 33 in different stages of colon cancer.

As shown in Figure 11, the levels of IL8 and IL33 are elevated in the early stages of colon cancer, with the highest values observed in stage 1 and stage 2, respectively. On the other hand, IL17A exhibits a peak in stage 2, followed by a significant decrease in stage 3 and 4.

### 3.2. Correlations between Interleukins for Healthy and Cancer Subjects

**Figure 12:**
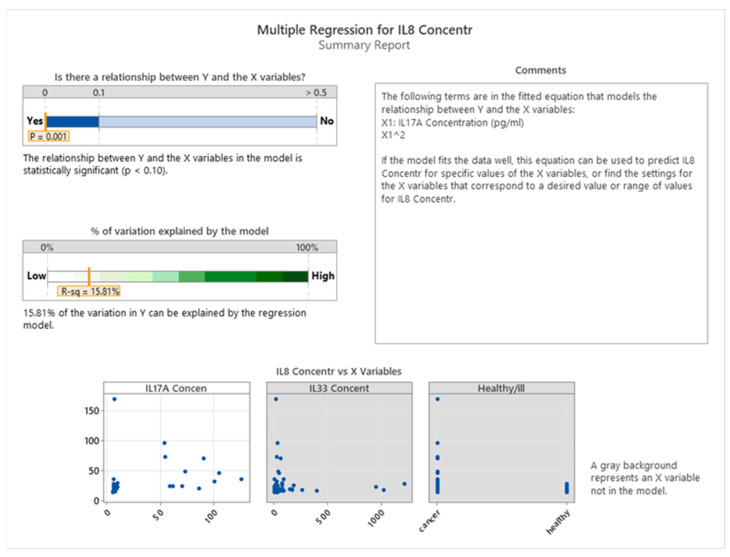
Influence of IL8 on IL17A and IL33 in healthy and cancer subjects.

Figure 12 shows there is a significant association between IL8 and IL17A levels in both healthy and cancer groups (*p* = 0.001), indicating that IL 17A may have an effect on IL8. However, no association is observed between IL8 and IL33 levels in either healthy or cancer patients.

**Figure 13:**
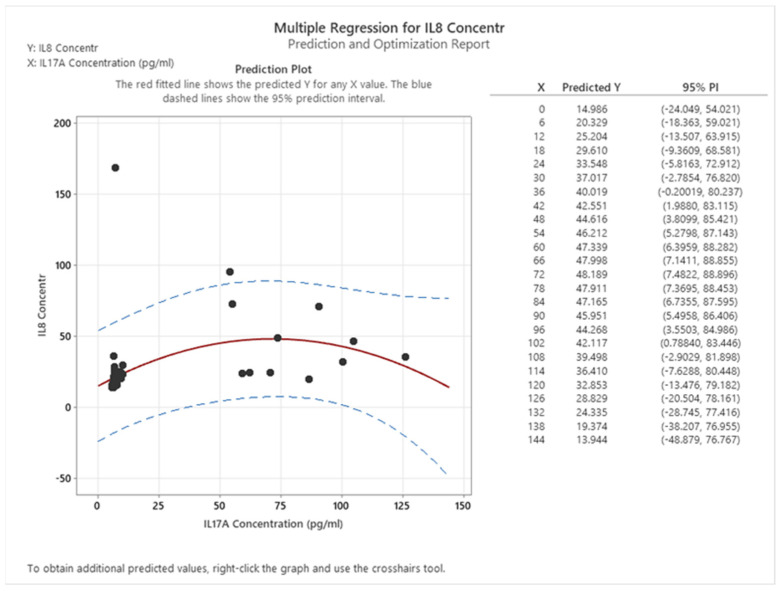
Correlation between IL8 and IL17 in healthy and colon cancer patients.

The presence of a statistically significant linear correlation between IL8 and IL17A is not observed. However, a quadratic correlation exists between these two interleukins in both healthy individuals and cancer patients. In particular, IL8 and IL17A exhibit a positive correlation up to a concentration of 75 pg/mL of IL17A, beyond which a negative correlation is observed (see Figure 13).

**Figure 14:**
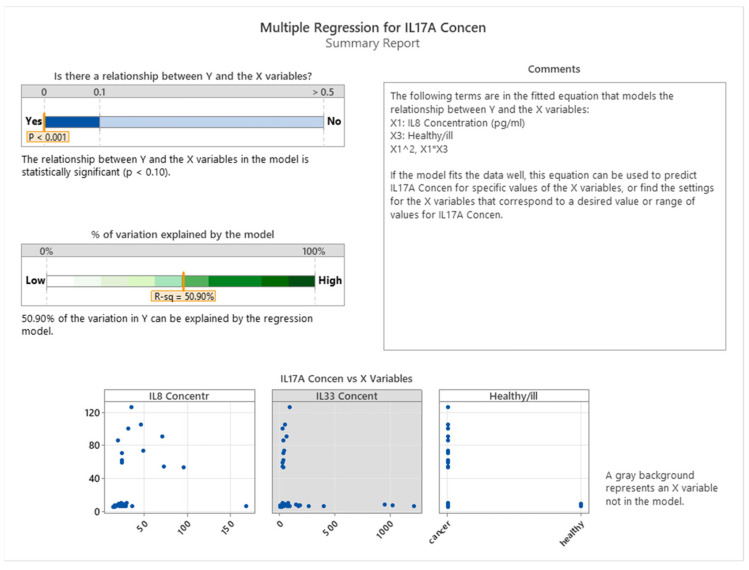
Influence of IL17A on IL8 and IL33 in healthy and cancer subjects.

We aimed to investigate the relationship between IL17A levels and IL8 and IL33 and the health status of the patients. The data showed a strong positive association between IL17A and IL8 (*p* < 0.001), implying that IL8 may regulate IL17A production. Furthermore, we found that the health status of the patients affected IL17A levels, as depicted in Figure 14. On the other hand, there was no evidence of any link between IL17A and IL33, suggesting that IL33 does not modulate IL17A in any manner. We measured the concentrations of these three interleukins in both healthy individuals and cancer patients.

**Figure 15:**
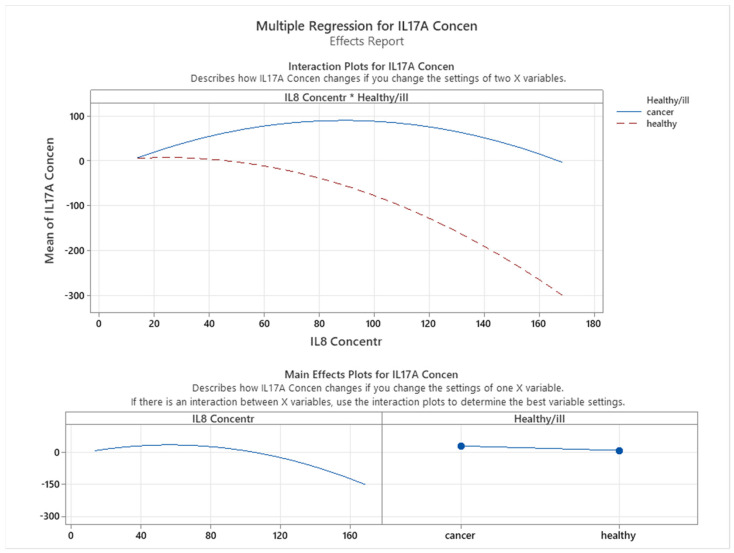
Correlation between IL8 and IL17A.

As shown in Figure 15, the relationship between interleukin 17A and IL8 varies depending on the health status of the patients. In cancer patients, there is a weak quadratic correlation between the two interleukins, suggesting that IL8 may have a complex effect on interleukin 17A production. In contrast, in healthy patients, we found a negative correlation between IL8 and interleukin 17A, indicating that higher levels of IL8 are associated with lower levels of interleukin 17A. Moreover, we observed that cancer patients had higher interleukin 17A levels than healthy patients.

**Figure 16:**
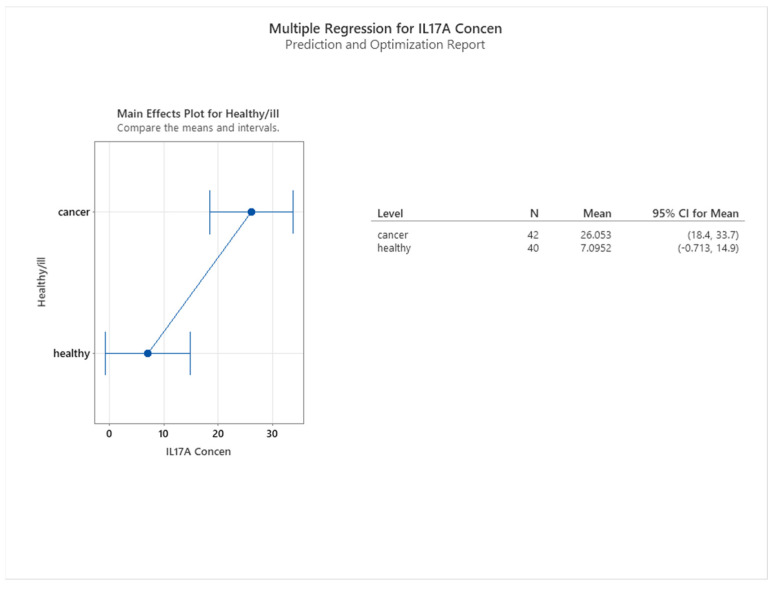
Correlation between IL17 concentration and health status of subjects.

**Figure 17:**
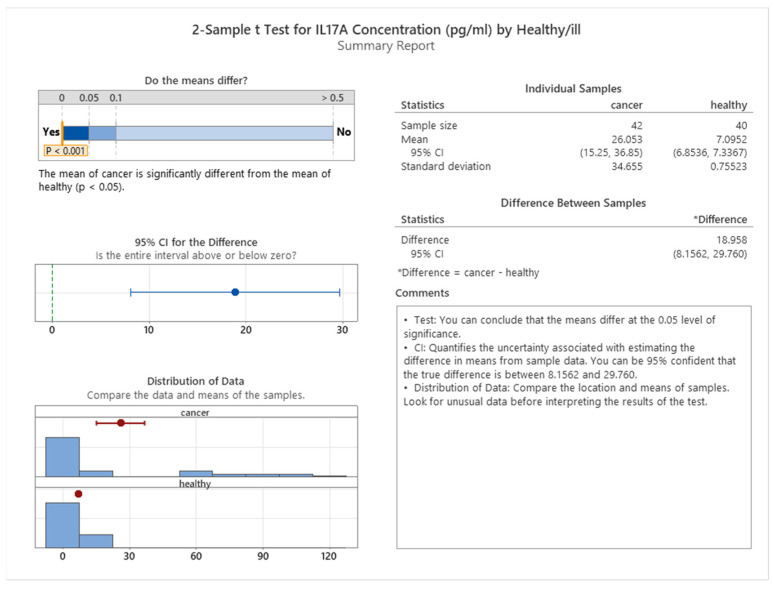
2-sample *t* test for IL17A by the health status of subjects.

Our study revealed a significant association between IL17A levels and health status. The median IL17A level in healthy participants was 7.09 pg/mL, whereas the median level in cancer patients was 26.05 pg/mL, indicating that IL17A is a marker of cancer risk (see Figure 16). We performed a 2-sample *t* test to compare the two groups and found a statistically significant difference (*p* < 0.001), as shown in Figure 17.

### 3.3. Interleukin Correlation in Cancer Patients

**Figure 18:**
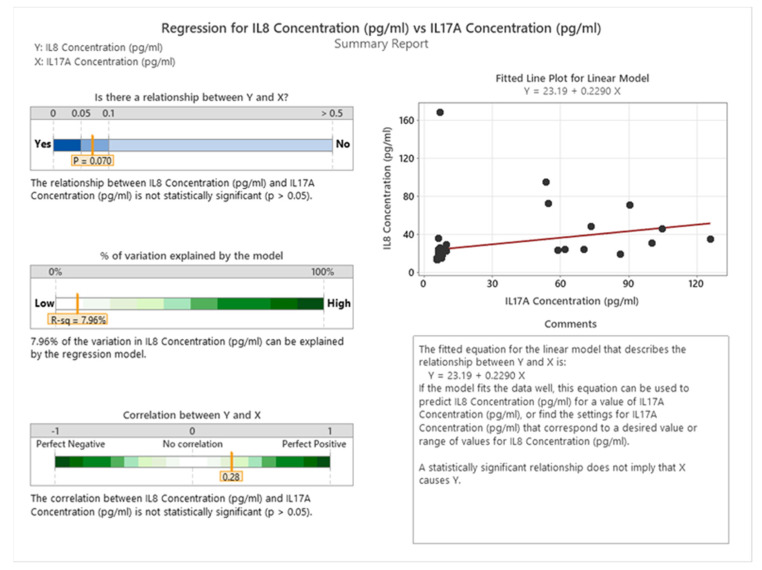
Correlation between IL8 and IL17A in cancer patients.

Figure 18 shows a weak positive relationship between IL8 and IL17A levels in cancer patients. This means that as IL8 increases, IL17A also tends to increase. The correlation coefficient is 0.28, which is close to zero, indicating a weak linear correlation. The *p*-value is 0.070, which is greater than the significance level of 0.05, meaning that the correlation is not statistically significant. Therefore, we cannot reject the null hypothesis that there is no linear correlation between IL8 and IL17A in cancer patients.

**Figure 19:**
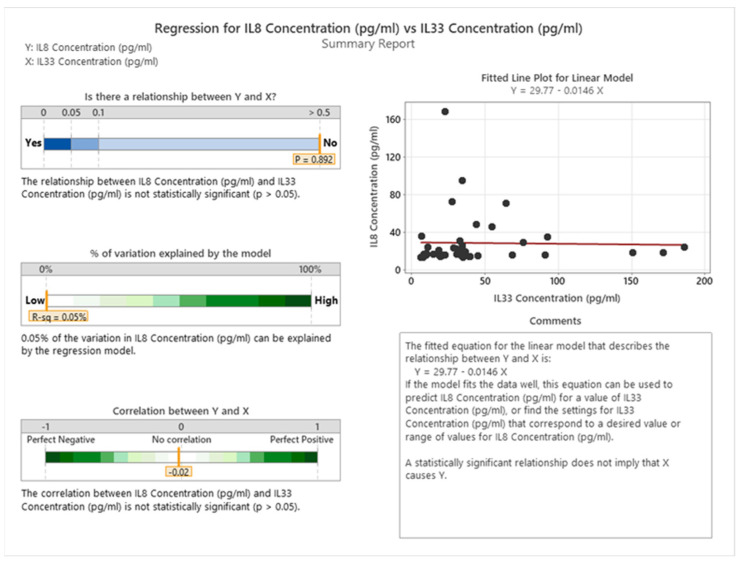
Correlation between IL8 and IL33 in cancer patients.

Our analysis did not reveal any significant associations between the levels of IL8 and IL33 in cancer patients. The correlation coefficient was −0.02, indicating a very weak negative correlation, and the *p*-value was 0.892, indicating a high probability of obtaining such results by chance. Therefore, we cannot infer any causal or functional relationship between the two interleukins based on our data (see Figure 19 for details).

**Figure 20:**
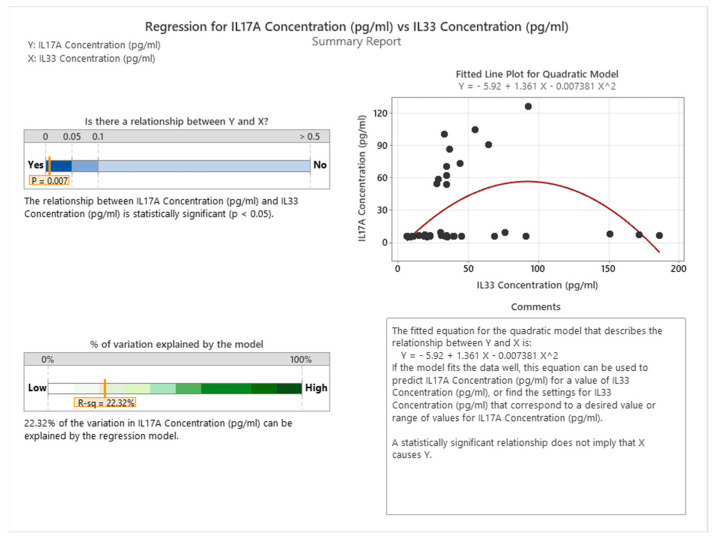
Correlation between IL17A and IL33 in cancer patients.

We demonstrated a statistically significant quadratic correlation between IL17A and IL33 (*p* = 0.007 < 0.05) in cancer patients, which means that, for IL33 concentrations below 100 pg/mL, IL17A concentration increases and as the IL33 concentration increases above this level, the IL17A concentration drops, as indicated in Figure 20.

### 3.4. Correlations between Interleukins in Different Cancer Stages

**Figure 21:**
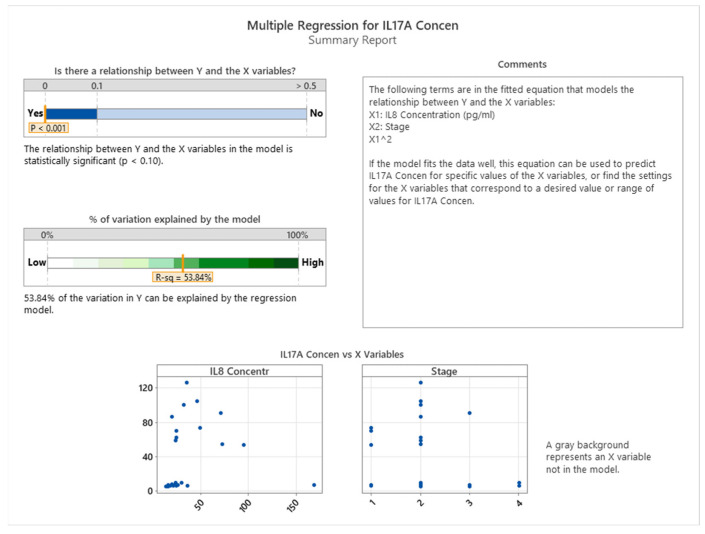
Correlation between IL17A and IL8 in different stages of colon cancer.

We observed statistically significant correlations between IL17A and IL8 in different stages of colon cancer (*p* < 0.001), as depicted in Figure 21.

**Figure 22:**
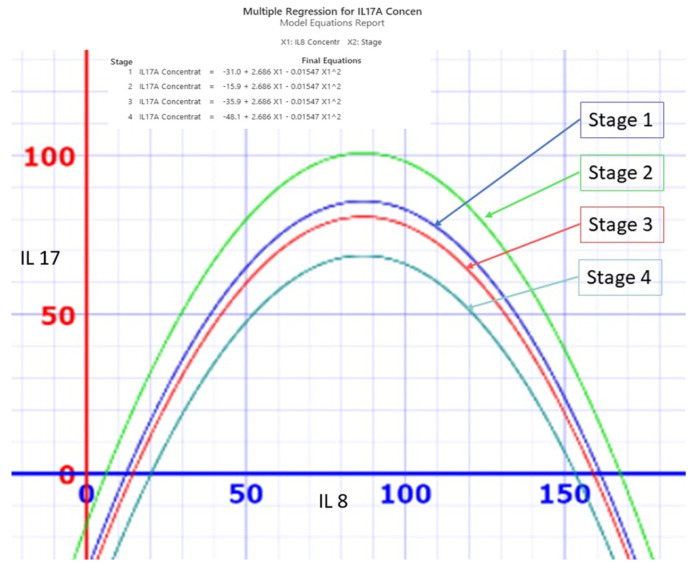
Correlation between IL17A and IL8 in the 4 stages of colon cancer.

The levels of IL17A and IL8, two cytokines involved in inflammation, vary across the four stages of colon cancer. The relationship between these two cytokines can be described by a quadratic function for each stage of the disease. As shown in Figure 22, the patients with stage 1 and 2 colon cancer have higher amounts of IL17A than those with stage 3 and 4. Moreover, IL17A and IL8 are positively correlated when IL8 is below 100 pg/mL, but they become negatively correlated when IL8 exceeds this threshold.

**Figure 23:**
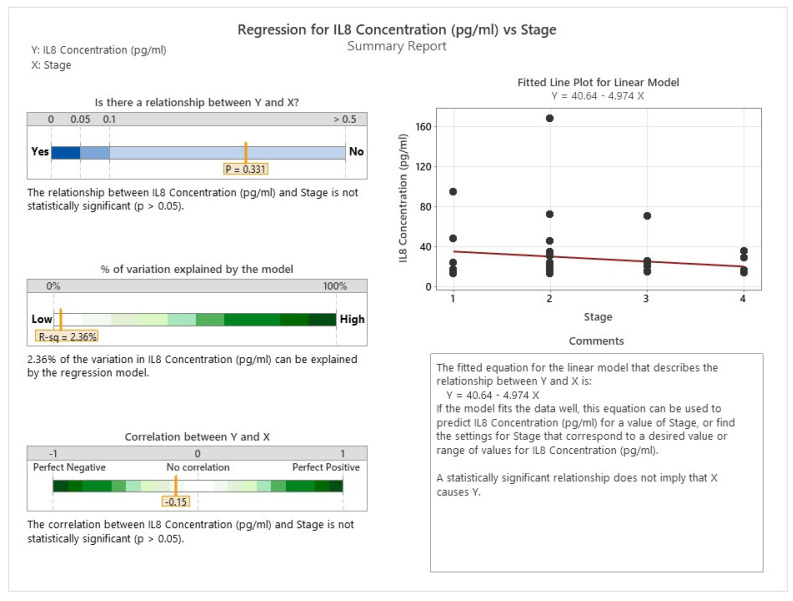
Correlation between IL8 and cancer staging.

**Figure 24:**
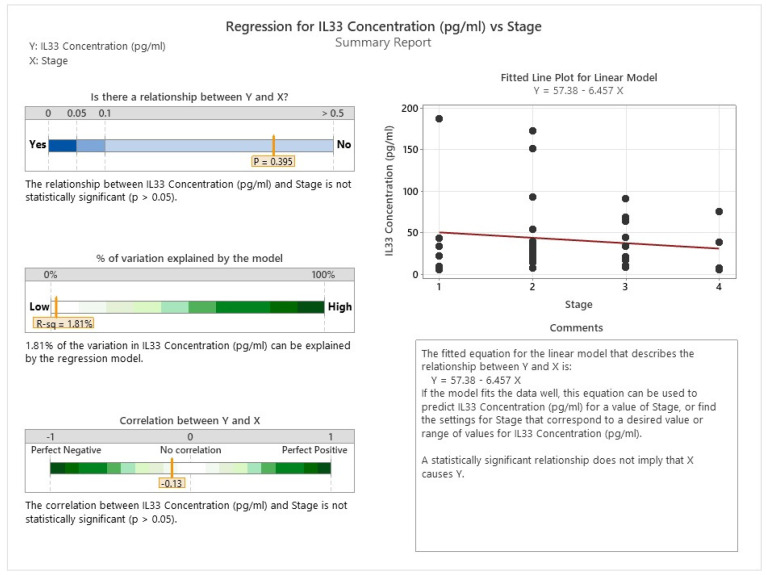
Correlation between IL33 and cancer staging.

As shown in Figure 23 and Figure 24, there is no evidence of any significant association between the levels of IL 8 and IL33 and the stage of cancer in the patients.

## 4. Discussion

Several studies have acknowledged the fact that cytokines from the tumour environment are involved in colon cancer progression as well as metastasis and that inflammation and cancer development are interdependent [30,31]. The main purpose of this study was to evaluate serum concentration of IL8, 17A, and IL33 and to establish correlations among these interleukins in different stages of colon cancer.

Colorectal cancer incidence varies throughout the world between different age groups, genders, and regions [32]. However, incidence of this malignancy in 50-year-old patients has been increasing over the past decade [33]. Up to one quarter of all patients already have metastatic disease at the time of diagnosis [34]. That is why early diagnosis and treatment is important for a better outcome of this disease. In our study, descriptive analysis showed associations between cancer staging, gender, and age. Our study showed that the peak in colon cancer incidence was at 68 years of age in a group of patients between 42 and 86 years. Studies show that the average age of colorectal cancer onset declined in the last 25 years from 67 to 61 years [35]. However, there is a low risk of developing colon cancer after 75 years of age if prior colonoscopies have been negative [36]. More female patients were diagnosed with stage 1 colon cancer, while men presented stage 2 and 3 colon cancer in this study. The stage 4 colon cancer group contained only men, emphasising that colon cancer is diagnosed later in men than women. Studies show higher mortality and lower 5-year survival rates in women over 65 years of age [37] and that women with colorectal cancer are 20% fewer than men [38]. It has been shown that younger women with metastatic disease have better survival rates than men of the same age, whereas older women have lower survival rates than older men with metastatic disease [39]. Overall, colon cancer incidence is significantly higher among men than women [40,41,42]. Correlations between stage and age showed that stage 1 and 3 colon cancer was more frequent in the 60–69 age group, while stage 2 cancer appeared in older patients. Most patients have been diagnosed with stage 2 cancer in our study. Studies also show an increase in stage 1 and 2 cancer rates, a decrease in stage 4 cancer rates [43], and a high incidence of stage 2 cancer in the elderly (70–79 years of age) [44]. Overall, our study showed that age and gender do not significantly influence colon cancer progression, as we did not find important correlations between colon cancer staging, age, or gender. Concerning interleukin concentrations, the highest for IL17A and IL33 was in the 60–69 age group, decreasing afterwards, while IL8’s highest concentration was between 60 and 79 years of age. Interleukin concentration was overall low in older age groups. Other studies have also shown a decrease in serum concentrations of interleukins, demonstrating a deterioration of the anti-inflammatory response associated with aging [45]. Although our study included more women than men, serum cytokine concentrations were significantly higher in the latter group, sustaining the literature data that the proinflammatory cytokine response is enhanced in men compared with women [46]. However, an important finding in our study is that interleukin concentrations are the highest in early stages of colon cancer, stating that inflammatory cytokines may count as biomarkers of early cancer stages, directly linking inflammation to cancer. It has been suggested by other studies that systemic inflammation might be linked to early development of colorectal cancer [47]. Also, high circulating proinflammatory cytokine levels in older patients with neoplasms may predict early mortality [48]. In our study, IL8 and IL17A were found to be higher in the cancer group, while IL33 concentration was higher in the control group, reinforcing the fact that inflammatory cytokines and colon cancer are correlated. The association between IL8 and 17A in colon cancer has already been investigated [15,49], but the role of IL33 in colon cancer still needs to be determined [50]. A correlation between IL33 and colorectal cancer progression has already been suggested [51].

Concerning interleukin correlations in healthy and cancer patients, we observed a statistically significant interdependence between IL8 and IL17A (*p* = 0.001) in the two groups, one strongly influencing the other’s response. As far as we know, this is the first study to investigate circulating IL8 and IL17A correlations in colon cancer. We found no statistically significant correlation between IL8 and IL33 or IL17A and IL33 between the control and cancer groups. In cancer patients, we observed a weaker correlation between IL8 and IL17A; however, there is a square correlation to be noticed between IL17A and IL33 (this study leaning to a direct response of circulating IL33 to IL17A variations in colon cancer development). We also observed a correlation between IL8 and IL17A, one determining the other’s response, in different stages of colon cancer, but no other correlations have been observed between other interleukins in the four stages of this malignancy.

## 5. Conclusions

This study aimed to evaluate the serum levels of IL8, IL17A, and IL33 and their correlations with different stages of colon cancer. The results showed that these cytokines, which are involved in inflammation and cancer development, were higher in the early stages of the disease, suggesting their potential as biomarkers for early detection and prognosis. The results also revealed that age and gender influenced the cytokine levels, with men having higher levels than women and older age groups having lower levels than younger ones. These findings are consistent with previous studies that have reported gender and age differences in colorectal cancer incidence, survival, and inflammatory response. This study contributes to the existing knowledge on the role of cytokines in colon cancer and provides insights for future research and clinical practice.

High levels of proinflammatory cytokines IL8 and 17A may count as biomarkers in early colon cancer stages, linking inflammation to neoplasia. Interleukin 8 and 17A and their associated expression levels may serve as predictive biomarkers of colorectal cancer. Larger groups of patients will be needed in order to confirm these hypotheses.

This study revealed the associations between serum levels of IL8, IL17A, and IL33 and colon cancer stages, as well as the correlations between these interleukins in healthy and cancer groups. However, the underlying mechanisms of how these interleukins regulate colon cancer progression and prognosis remain unclear. Therefore, future studies should consider measuring the mRNA levels of these interleukins in the same patient groups to correlate the mRNA expression and the protein levels of the ILs. This could provide more insights into the transcriptional and post-transcriptional regulation of these cytokines in colon cancer. Moreover, future studies should also investigate the functional roles of these interleukins in colon cancer cells and the tumour microenvironment, such as their effects on cell proliferation, apoptosis, invasion, angiogenesis, and immune response. This could elucidate the molecular pathways and targets of these cytokines in colon cancer development and therapy. Additionally, future studies should explore the potential of these interleukins as biomarkers or therapeutic agents for colon cancer, by validating their diagnostic and prognostic value, as well as their efficacy and safety in clinical trials. This could facilitate the early detection and personalised treatment of colon cancer.

## 6. Study Limitations

This study has some limitations that should be acknowledged. First, the sample size of 82 participants may not be sufficient to capture the full diversity and complexity of the population of interest. However, the sample size was determined by a power analysis based on previous studies and was adequate to detect significant differences and correlations between the groups [52]. Second, this study only measured the serum levels of the interleukins and did not examine their mRNA expression or their functional roles in colon cancer cells and the tumour microenvironment [53]. Therefore, this study could not explain the underlying mechanisms of how these interleukins regulate colon cancer progression and prognosis. Third, this study used the ELISA test to measure the serum levels of the interleukins, which may have some measurement errors or variability. The ELISA test is a widely used and validated method, but it may not be the most accurate or sensitive technique to quantify the cytokines [54]. Fourth, this study did not control for potential confounding variables that may affect the serum levels of the interleukins, such as age, gender, lifestyle, medication, comorbidities, and inflammation [55]. These factors may influence the results and limit the generalisability of the findings.

## Figures and Tables

**Table 1:** IL 8, 17, and 33 mean concentration, SE, and SD by age groups. * the number of missing values in each age group.

Variable	Age Group	N	N *	Mean	SE Mean	StDev	Minimum	Q1	Median	Q3	Maximum
IL8 Concentration (pg/mL)	40–49	3	0	15.85	1.26	2.19	13.78	13.78	15.62	18.14	18.14
	50–59	6	0	18.87	2.09	5.11	13.78	15.16	16.65	24.82	26.18
	60–69	14	0	36.19	6.62	24.75	15.62	21.01	24.70	44.34	95.34
	70–79	11	0	37.9	13.6	45.2	13.8	16.3	19.1	46.4	168.6
	80–89	8	0	17.51	1.15	3.25	14.93	15.04	16.30	20.50	23.42
IL17A Concentration (pg/mL)	40–49	3	0	6.073	0.189	0.327	5.801	5.801	5.982	6.436	6.436
	50–59	6	0	15.69	9.29	22.76	5.80	5.94	6.57	20.84	62.14
	60–69	14	0	43.5	11.1	41.6	6.3	7.2	32.0	75.5	126.0
	70–79	11	0	28.9	11.7	38.8	6.3	6.4	6.5	73.6	104.8
	80–89	8	0	6.957	0.461	1.304	6.254	6.277	6.436	7.138	10.062
IL33 Concentration (pg/mL)	40–49	3	0	21.98	7.17	12.42	10.17	10.17	20.84	34.93	34.93
	50–59	6	0	32.0	12.8	31.4	8.1	8.8	24.6	48.8	91.2
	60–69	14	0	60.5	14.0	52.4	10.9	25.5	34.5	80.3	186.5
	70–79	11	0	39.8	14.0	46.6	6.1	10.0	23.0	44.0	171.9
	80–89	8	0	30.16	4.07	11.52	8.22	22.39	32.09	38.14	45.25

**Table 2:** IL 8, 17, and 33 mean concentration, SE, and SD by gender. * the number of missing values in each age group.

Variable	Gender	N	N *	Mean	SE Mean	StDev	Minimum	Q1	Median	Q3	Maximum
IL8 Concentration (pg/mL)	F	23	0	21.07	1.68	8.07	13.78	16.08	17.22	24.37	46.40
	M	19	0	38.94	8.99	39.20	13.78	15.62	21.59	49.10	168.56
IL17A Concentration (pg/mL)	F	23	0	20.06	6.51	31.22	5.80	6.34	6.44	7.34	104.79
	M	19	0	33.31	8.71	37.98	5.98	6.53	8.25	59.05	126.00
IL33 Concentration (pg/mL)	F	23	0	34.90	8.17	39.17	6.13	10.17	23.01	34.68	186.48
	M	19	0	51.6	10.1	43.8	9.0	27.7	34.9	64.4	171.9

**Table 3:** IL 8, 17, and 33 mean concentration, SE, and SD by age group for cancer patients. * the number of missing values in each group.

Variable	Age Group	N	N *	Mean	SE Mean	StDev	Minimum	Q1	Median	Q3	Maximum
IL8 Concentration (pg/mL)	40–49	3	0	15.85	1.26	2.19	13.78	13.78	15.62	18.14	18.14
	50–59	6	0	18.87	2.09	5.11	13.78	15.16	16.65	24.82	26.18
	60–69	14	0	36.19	6.62	24.75	15.62	21.01	24.70	44.34	95.34
	70–79	11	0	37.9	13.6	45.2	13.8	16.3	19.1	46.4	168.6
	80–89	8	0	17.51	1.15	3.25	14.93	15.04	16.30	20.50	23.42
IL17A Concentration (pg/mL)	40–49	3	0	6.073	0.189	0.327	5.801	5.801	5.982	6.436	6.436
	50–59	6	0	15.69	9.29	22.76	5.80	5.94	6.57	20.84	62.14
	60–69	14	0	43.5	11.1	41.6	6.3	7.2	32.0	75.5	126.0
	70–79	11	0	28.9	11.7	38.8	6.3	6.4	6.5	73.6	104.8
	80–89	8	0	6.957	0.461	1.304	6.254	6.277	6.436	7.138	10.062
IL33 Concentration (pg/mL)	40–49	3	0	21.98	7.17	12.42	10.17	10.17	20.84	34.93	34.93
	50–59	6	0	32.0	12.8	31.4	8.1	8.8	24.6	48.8	91.2
	60–69	14	0	60.5	14.0	52.4	10.9	25.5	34.5	80.3	186.5
	70–79	11	0	39.8	14.0	46.6	6.1	10.0	23.0	44.0	171.9
	80–89	8	0	30.16	4.07	11.52	8.22	22.39	32.09	38.14	45.25

**Table 4:** IL 8, 17, and 33 mean concentration, SE, and SD by age group for healthy patients. * the number of missing values in each group.

Variable	Age Group	N	N *	Mean	SE Mean	StDev	Minimum	Q1	Median	Q3	Maximum
IL8 Concentration (pg/mL)	40–49	2	0	22.73	2.53	3.57	20.21	*	22.73	*	25.26
	50–59	14	0	18.70	1.07	4.00	14.24	15.39	17.45	20.67	27.56
	60–69	8	0	19.606	0.989	2.796	15.616	17.166	19.520	22.448	23.194
	70–79	9	0	19.93	1.31	3.94	15.62	17.22	18.60	22.39	28.48
	80–89	7	0	17.551	0.549	1.452	15.386	16.305	17.912	19.060	19.290
IL17A Concentration (pg/mL)	40–49	2	0	7.750	0.408	0.577	7.342	*	7.750	*	8.158
	50–59	14	0	7.232	0.275	1.030	6.073	6.640	6.844	8.068	9.337
	60–69	8	0	7.127	0.255	0.720	6.073	6.730	6.934	7.864	8.249
	70–79	9	0	6.859	0.113	0.339	6.436	6.617	6.798	6.980	7.614
	80–89	7	0	6.902	0.200	0.530	5.982	6.526	6.889	7.433	7.524
IL33 Concentration (pg/mL)	40–49	2	0	17.848	0.755	1.068	17.093	*	17.848	*	18.603
	50–59	14	0	149.6	86.9	325.2	6.0	7.7	24.6	104.4	1217.1
	60–69	8	0	144	116	327	7	12	23	71	951
	70–79	9	0	52.6	27.2	81.7	6.7	10.5	20.7	56.6	262.8
	80–89	7	0	158	144	380	7	7	9	35	1019

## Data Availability

The data presented in this study are available on request from the corresponding author.

## References

[1] Ning Y., Lenz H.-J. (2012). Targeting IL-8 in Colorectal Cancer. Expert Opin. Ther. Targets.

[2] Gefen R., Emile S.H., Horesh N., Garoufalia Z., Wexner S.D. (2023). Age-Related Variations in Colon and Rectal Cancer: An Analysis of the National Cancer Database. Surgery.

[3] Wong M.C.S., Huang J., Lok V., Wang J., Fung F., Ding H., Zheng Z.-J. (2021). Differences in Incidence and Mortality Trends of Colorectal Cancer Worldwide Based on Sex, Age, and Anatomic Location. Clin. Gastroenterol. Hepatol. Off. Clin. Pract. J. Am. Gastroenterol. Assoc..

[4] Su Y., Tian X., Gao R., Guo W., Chen C., Chen C., Jia D., Li H., Lv X. (2022). Colon Cancer Diagnosis and Staging Classification Based on Machine Learning and Bioinformatics Analysis. Comput. Biol. Med..

[5] Pilleron S., Withrow D.R., Nicholson B.D., Morris E.J.A. (2023). Age-Related Differences in Colon and Rectal Cancer Survival by Stage, Histology, and Tumour Site: An Analysis of United States SEER-18 Data. Cancer Epidemiol..

[6] Lin L., Xu W., Zhang G., Ren P., Zhao J., Yan Q. (2017). Association of Interleukin-22 Polymorphisms with the Colon Cancer: A Case-Control Study. Immunol. Lett..

[7] Rebeneck L., Horton S., Zauber A.G., Eerle C. Colorectal Cancer. https://pubmed.ncbi.nlm.nih.gov/26913342/.

[8] Sharma B.R., Kanneganti T.-D. (2023). Inflammasome Signaling in Colorectal Cancer. Transl. Res. J. Lab. Clin. Med..

[9] Terzić J., Grivennikov S., Karin E., Karin M. (2010). Inflammation and Colon Cancer. Gastroenterology.

[10] Moossavi M., Parsamanesh N., Bahrami A., Atkin S.L., Sahebkar A. (2018). Role of the NLRP3 Inflammasome in Cancer. Mol. Cancer.

[11] Hamarsheh S., Zeiser R. (2020). NLRP3 Inflammasome Activation in Cancer: A Double-Edged Sword. Front. Immunol..

[12] Quagliariello V., Passariello M., Di Mauro A., Cipullo C., Paccone A., Barbieri A., Palma G., Luciano A., Buccolo S., Bisceglia I. (2022). Immune Checkpoint Inhibitor Therapy Increases Systemic SDF-1, Cardiac DAMPs Fibronectin-EDA, S100/Calgranulin, Galectine-3, and NLRP3-MyD88-Chemokine Pathways. Front. Cardiovasc. Med..

[13] Li J., Huang L., Zhao H., Yan Y., Lu J. (2020). The Role of Interleukins in Colorectal Cancer. Int. J. Biol. Sci..

[14] Joshi S., Pandey R., Kumar A., Gupta V., Arya N. (2023). Targeted Blockade of Interleukin-8 Negates Metastasis and Chemoresistance via Akt/Erk-NFκB Axis in Oral Cancer. Cytokine.

[15] Bazzichetto C., Milella M., Zampiva I., Simionato F., Amoreo C.A., Buglioni S., Pacelli C., Le Pera L., Colombo T., Bria E. (2022). Interleukin-8 in Colorectal Cancer: A Systematic Review and Meta-Analysis of Its Potential Role as a Prognostic Biomarker. Biomedicines.

[16] Cacev T., Radosević S., Krizanac S., Kapitanović S. (2008). Influence of Interleukin-8 and Interleukin-10 on Sporadic Colon Cancer Development and Progression. Carcinogenesis.

[17] Iwakura Y., Ishigame H., Saijo S., Nakae S. (2011). Functional Specialization of Interleukin-17 Family Members. Immunity.

[18] Wu D., Wu P., Huang Q., Liu Y., Ye J., Huang J. (2013). Interleukin-17: A Promoter in Colorectal Cancer Progression. Clin. Dev. Immunol..

[19] Razi S., Baradaran Noveiry B., Keshavarz-Fathi M., Rezaei N. (2019). IL-17 and Colorectal Cancer: From Carcinogenesis to Treatment. Cytokine.

[20] Bedoui S.A., Barbirou M., Stayoussef M., Dallel M., Mokrani A., Makni L., Mezlini A., Bouhaouala B., Yacoubi-Loueslati B., Almawi W.Y. (2018). Association of Interleukin-17A Polymorphisms with the Risk of Colorectal Cancer: A Case-Control Study. Cytokine.

[21] Cayrol C., Girard J.-P. (2018). Interleukin-33 (IL-33): A Nuclear Cytokine from the IL-1 Family. Immunol. Rev..

[22] Xu H., Turnquist H.R., Hoffman R., Billiar T.R. (2017). Role of the IL-33-ST2 Axis in Sepsis. Mil. Med. Res..

[23] Quagliariello V., Paccone A., Iovine M., Cavalcanti E., Berretta M., Maurea C., Canale M., Maurea N. (2021). Interleukin-1 Blocking Agents as Promising Strategy for Prevention of Anticancer Drug-Induced Cardiotoxicities: Possible Implications in Cancer Patients with COVID-19. Eur. Rev. Med. Pharmacol. Sci..

[24] Zhang Y., Davis C., Shah S., Hughes D., Ryan J.C., Altomare D., Peña M.M.O. (2017). IL-33 Promotes Growth and Liver Metastasis of Colorectal Cancer in Mice by Remodeling the Tumor Microenvironment and Inducing Angiogenesis. Mol. Carcinog..

[25] Chen X., Lu K., Timko N.J., Weir D.M., Zhu Z., Qin C., Mann J.D., Bai Q., Xiao H., Nicholl M.B. (2018). IL-33 Notably Inhibits the Growth of Colon Cancer Cells. Oncol. Lett..

[26] Jou E., Rodriguez-Rodriguez N., McKenzie A.N.J. (2022). Emerging Roles for IL-25 and IL-33 in Colorectal Cancer Tumorigenesis. Front. Immunol..

[27] Zhao J., Chen X., Herjan T., Li X. (2019). The Role of Interleukin-17 in Tumor Development and Progression. J. Exp. Med..

[28] Colorectal Cancer Stages. Rectal Cancer Staging. Colon Cancer Staging. https://www.cancer.org/cancer/types/colon-rectal-cancer/detection-diagnosis-staging/staged.html.

[29] Karamchandani D.M., Chetty R., King T.S., Liu X., Westerhoff M., Yang Z., Yantiss R.K., Driman D.K. (2020). Challenges with Colorectal Cancer Staging: Results of an International Study. Mod. Pathol..

[30] Cui G., Liu H., Laugsand J.-B. (2023). Endothelial Cells-Directed Angiogenesis in Colorectal Cancer: Interleukin as the Mediator and Pharmacological Target. Int. Immunopharmacol..

[31] Knüpfer H., Preiss R. (2010). Serum Interleukin-6 Levels in Colorectal Cancer Patients—A Summary of Published Results. Int. J. Colorectal Dis..

[32] Baidoun F., Elshiwy K., Elkeraie Y., Merjaneh Z., Khoudari G., Sarmini M.T., Gad M., Al-Husseini M., Saad A. (2021). Colorectal Cancer Epidemiology: Recent Trends and Impact on Outcomes. Curr. Drug Targets.

[33] Patel S.G., Karlitz J.J., Yen T., Lieu C.H., Boland C.R. (2022). The Rising Tide of Early-Onset Colorectal Cancer: A Comprehensive Review of Epidemiology, Clinical Features, Biology, Risk Factors, Prevention, and Early Detection. Lancet Gastroenterol. Hepatol..

[34] Haraldsdottir S., Einarsdottir H.M., Smaradottir A., Gunnlaugsson A., Halfdanarson T.R. (2014). Colorectal cancer—Review. Laeknabladid.

[35] Liu A., Zheng Y., Yang P., Chu H., Hou X. (2023). Change in Onset Age of First Primary Colorectal Cancer in the USA. Int. J. Colorectal Dis..

[36] Lieberman D. (2023). At What Age Should We Stop Colorectal Cancer Screening? When Is Enough, Enough?. Cancer Epidemiol. Biomark. Prev. Publ. Am. Assoc. Cancer Res. Cosponsored Am. Soc. Prev. Oncol..

[37] Kim S.-E., Paik H.Y., Yoon H., Lee J.E., Kim N., Sung M.-K. (2015). Sex- and Gender-Specific Disparities in Colorectal Cancer Risk. World J. Gastroenterol..

[38] Hultcrantz R. (2021). Aspects of Colorectal Cancer Screening, Methods, Age and Gender. J. Intern. Med..

[39] Hendifar A., Yang D., Lenz F., Lurje G., Pohl A., Lenz C., Ning Y., Zhang W., Lenz H.-J. (2009). Gender Disparities in Metastatic Colorectal Cancer Survival. Clin. Cancer Res. Off. J. Am. Assoc. Cancer Res..

[40] Van Erning F.N., Greidanus N.E.M., Verhoeven R.H.A., Buijsen J., de Wilt H.W., Wagner D., Creemers G.-J. (2023). Gender Differences in Tumor Characteristics, Treatment and Survival of Colorectal Cancer: A Population-Based Study. Cancer Epidemiol..

[41] Ribbing Wilén H., Saraste D., Blom J. (2021). Gender-Specific Cut-off Levels in Colorectal Cancer Screening with Fecal Immunochemical Test: A Population-Based Study of Colonoscopy Findings and Costs. J. Med. Screen..

[42] Gao R.-N., Neutel C.I., Wai E. (2008). Gender Differences in Colorectal Cancer Incidence, Mortality, Hospitalizations and Surgical Procedures in Canada. J. Public Health Oxf. Engl..

[43] Zhylkaidarova A., Kaidarova D., Batyrbekov K., Shatkovskaya O., Begimbetova D. (2021). Trends of Colorectal Cancer Prevalence in Kazakhstan Related to Screening. Clin. Endosc..

[44] Ullah M.F., Fleming C.A., Mealy K. (2018). Changing Trends in Age and Stage of Colorectal Cancer Presentation in Ireland—From the Nineties to Noughties and Beyond. Surg. J. R. Coll. Surg. Edinb. Irel..

[45] Ito H., Miki C. (1999). Profile of Circulating Levels of Interleukin-1 Receptor Antagonist and Interleukin-6 in Colorectal Cancer Patients. Scand. J. Gastroenterol..

[46] Heesen M., Bloemeke B., Heussen N., Kunz D. (2002). Can the Interleukin-6 Response to Endotoxin Be Predicted? Studies of the Influence of a Promoter Polymorphism of the Interleukin-6 Gene, Gender, the Density of the Endotoxin Receptor CD14, and Inflammatory Cytokines. Crit. Care Med..

[47] Kim S., Keku T.O., Martin C., Galanko J., Woosley J.T., Schroeder J.C., Satia J.A., Halabi S., Sandler R.S. (2008). Circulating Levels of Inflammatory Cytokines and Risk of Colorectal Adenomas. Cancer Res..

[48] Kim S.H., Kim J.W., Hwang I.G., Jang J.S., Hong S., Kim T.-Y., Baek J.Y., Shin S.H., Sun D.S., Hong D.-S. (2019). Serum Biomarkers for Predicting Overall Survival and Early Mortality in Older Patients with Metastatic Solid Tumors. J. Geriatr. Oncol..

[49] Zarogoulidis P., Katsikogianni F., Tsiouda T., Sakkas A., Katsikogiannis N., Zarogoulidis K. (2014). Interleukin-8 and Interleukin-17 for Cancer. Cancer Investig..

[50] Huang F., Chen W.-Y., Ma J., He X.-L., Wang J.-W. (2022). Paradoxical Role of Interleukin-33/Suppressor of Tumorigenicity 2 in Colorectal Carcinogenesis: Progress and Therapeutic Potential. World J. Clin. Cases.

[51] Akimoto M., Takenaga K. (2019). Role of the IL-33/ST2L Axis in Colorectal Cancer Progression. Cell. Immunol..

[52] Borkowf C.B., Johnson L.L., Albert P.S. (2018). Power and Sample Size Calculations. Principles and Practice of Clinical Research.

[53] Wei X., Zhang Y., Yang Z., Sha Y., Pan Y., Chen Y., Cai L. (2020). Analysis of the Role of the Interleukins in Colon Cancer. Biol. Res..

[54] ELISAs for Quantifying Cytokine Release. https://www.rndsystems.com/products/cytokine-elisa-kits.

[55] Briukhovetska D., Dörr J., Endres S., Libby P., Dinarello C.A., Kobold S. (2021). Interleukins in Cancer: From Biology to Therapy. Nat. Rev. Cancer.

